# A systematic review of the status of children's school access in low- and middle-income countries between 1998 and 2013: using the INDEPTH Network platform to fill the research gaps

**DOI:** 10.3402/gha.v8.28430

**Published:** 2015-11-11

**Authors:** Mamusu Kamanda, Osman Sankoh

**Affiliations:** 1 Scientific Research and Co-ordination, INDEPTH Network, Accra, Ghana; 2 School of Public Health, Faculty of Health Sciences, University of Witwatersrand, Johannesburg, South Africa; 3 Faculty of Public Health, Hanoi Medical University, Hanoi, Vietnam

**Keywords:** school access, enrolment, attendance, grade progression, dropout, primary to secondary school transition, completion

## Abstract

**Background:**

The framework for expanding children's school access in low- and middle-income countries (LMICs) has been directed by universal education policies as part of Education for All since 1990. In measuring progress to universal education, a narrow conceptualisation of access which dichotomises children's participation as being in or out of school has often been assumed. Yet, the actual promise of universal education goes beyond this simple definition to include retention, progression, completion, and learning.

**Objective:**

Our first objective was to identify gaps in the literature on children's school access using the zones of exclusion of the Consortium for Research on Educational Access, Transition, and Equity as a framework. Second, we gave consideration to how these gaps can be met by using longitudinal and cross-country data from Health and Demographic Surveillance System (HDSS) sites within the International Network for the Demographic Evaluation of Population and Their Health (INDEPTH) in LMICs.

**Design:**

Using Web of Science, we conducted a literature search of studies published in international peer-reviewed journals between 1998 and 2013 in LMICs. The phrases we searched included six school outcomes: school enrolment, school attendance, grade progression, school dropout, primary to secondary school transition, and school completion. From our search, we recorded studies according to: 1) school outcomes; 2) whether longitudinal data were used; and 3) whether data from more than one country were analysed.

**Results:**

The area of school access most published is enrolment followed by attendance and dropout. Primary to secondary school transition and grade progression had the least number of publications. Of 132 publications which we found to be relevant to school access, 33 made use of longitudinal data and 17 performed cross-country analyses.

**Conclusions:**

The majority of studies published in international peer-reviewed journals on children's school access between 1998 and 2013 were focused on three outcomes: enrolment, attendance, and dropout. Few of these studies used data collected over time or data collected from more than one country for comparative analyses. The contribution of the INDEPTH Network in helping to address these gaps in the literature lies in the longitudinal design of HDSS surveys and in the diversity of countries within the network.

Since 1990, Education for All (EFA) has generated much research interest on children's school access in low- and middle-income countries (LMICs). EFA is a global policy framework designed to expand access to education among children. It was first introduced in 1990 at the World Conference on EFA in Jomtien, Thailand, specifying six goals to be achieved by 2000 (1). One of the goals, Universal Primary Education (UPE, Goal 2), has been the centrepiece for the EFA movement. The UPE promised to ‘ensure that by 2005 all children, particularly girls, children in difficult circumstances, and those belonging to ethnic minorities, have access to, and complete, free and compulsory primary education of good quality’ (2). In 2000, the policy was recommitted at the World Education Forum (WEF) in Dakar, Senegal (3). The goals outlined at the WEF were similar to those developed at the 1990 World Conference. Two of the EFA goals – UPE and Equal Gender Parity – were included as Goal 2 of the Millennium Development Goals (MDGs) in 2000. Through the MDGs, UPE continued to form the focus of international and national investment. The success of this investment led to the adoption of Universal Basic Education (UBE), a policy which extended the UBE policy by promising to provide universal primary and lower secondary education.

The core of UBE is to expand ‘access’ to school for children. Both the World Conference and WEF for the EFA however did not provide a strict definition of access. Rather, a series of indicators were designed to measure progress towards universal education. The most commonly used indicators have been the gross enrolment (GER)/attendance ratio (GAR) and the net enrolment (NER)/attendance ratio (NAR) (2). Enrolment ratios refer to the number of children who are enrolled in school. Attendance ratios, by comparison, refer not to enrolment but to the number of children attending school. The distinction is important because the two indicators can give very diverse implications of school participation. In many LMICs, it is not uncommon to have higher enrolment rates than attendance rates. The reason being that households may enrol a child but that child may have infrequent attendance or indeed may not attend school at all, meaning that participation rates may be inflated by enrolment rates. This problem is compounded by poor qualities of data collection and management systems, particularly in low-income settings, which make it difficult to capture actual rates of attendance and enrolment.

The use of the GER, GAR, NER, and NAR as indicators of progress towards universal education implied that ‘access’ was to be understood as the proportion of children who had gained admission to school. This in turn suggested a dichotomous definition of ‘access’ with: 1) children in the education system and 2) those ‘out-of-school’. With this narrow definition, the race to achieving UPE became concerned with reducing the ‘out-of-school’ population with little attention being paid to how children progressed in the education system once they entered school.

The model of educational access of the Consortium for Research on Educational Access, Transition, and Equity (CREATE) provides a broader conceptualisation of access by identifying zones of exclusion which highlight various patterns of school behaviour among children of school age (Table 1). Zone 0 includes children who are excluded from pre-school. Children who have never enrolled in primary school are captured by Zone 1. Zone 2 refers to children who have enrolled in primary school but who then subsequently dropout. Zone 3 covers a vulnerable section of the in-school population who are at an increased risk of dropping out. This group of children includes overage children, irregular attenders, and low achievers. Children who complete primary school but are unable to transition into lower secondary school form the focus of Zone 4. Zone 5 holds a similar definition to Zone 2 in its emphasis on school dropouts; it refers to children who enter secondary school but are unable to remain in school for the full secondary school cycle. The final zone covers the same group of children as in Zone 3 but at the secondary school level.

**Table 1:** Consortium for Research on Educational Access, Transitions and Equity's zones of exclusion for educational access among children of school age

Zones of exclusion	Description
Zone 0	No pre-school access
Zone 1	Children who never enrol in primary school
Zone 2	Primary dropouts
Zone 3	Overage children, irregular attenders, and low-achievers at primary level who are ‘silently excluded’ and learn little
Zone 4	Primary leavers not entering secondary
Zone 5	Secondary dropouts
Zone 6	Overage children, irregular attenders, low-achievers, and those silently excluded at secondary level

Definition taken from Lewin (4).

The zones of exclusion conceptualise access as a continuum of participation within an education system, entering at the pre-school level and remaining in school until the end of secondary education. It accounts for enrolment, attendance, progression, dropout, and transition from primary to secondary school, completion of a school cycle, and learning of the school curriculum. This definition better reflects the realities of school behaviour in LMICs and the promise of UBE contained in the EFA framework. That is, in order for education to be meaningfully universal, simply enrolling a child into school is inadequate. Upon entering school, children must be able to regularly attend school, move from one grade to the next, and complete a full course of primary education and lower secondary education. Furthermore, having completed a cycle of school, children should be able to demonstrate competence in the curriculum.

For this study, we use CREATE's zones of educational exclusion to review studies which have explored access to primary and secondary school between 1993 and 2013 in LMICs. Our aim was to identify gaps in the literature focusing on: 1) the least explored school outcomes; 2) studies using longitudinal data; and 3) studies performing cross-country analyses. We restricted our attention to studies that have explored school participation and so we only considered studies where the outcome was one or more of the following: school enrolment, school attendance, grade progression, school dropout, primary to lower secondary transition, and school completion. Having reviewed the literature, we move to discuss how data from Health and Demographic Surveillance Systems (HDSS) sites within the International Network for the Demographic Evaluation of Population and Their Health (INDEPTH) Network can be used to fill the evidence gaps that we identify through our review.

The INDEPTH is a not-for-profit non-governmental organization currently comprising 52 HDSS sites in 20 LMICs in Africa, Asia, and Oceania (5). The majority of the 52 HDSS sites collect routine information on children's current school status, individual as well as household-level demographic and socio-economic information, and school availability and type of school. Some sites use geographic information system to enumerate the number of schools by type. The HDSS sites continuously monitor and evaluate populations and their health over time. They survey mainly three types of populations in LMICs including those in: 1) rural areas; 2) border towns; and 3) urban informal settlements. Operating within the same local population over time, the HDSS sites are able to detect change at a microlevel in the dynamics of the population, including changes in children's schooling outcomes.

The longitudinal arrangement of the HDSS sites and the diversity of countries within the INDEPTH Network offer a unique opportunity to further explore children's access to school in LMICs through longitudinal analyses and cross-country analyses. Longitudinal data analyses can help us to better uncover the temporal pathways of children's transition through the education system and how these transitions may be affected by conditions within households and communities. We are also able to better observe changes in local conditions (i.e. political, economic, and demographic changes) and relate these contextual changes to children's school outcomes over time. Cross-country analyses as well as comparisons between multiple localities in a single country can help us to engage with more nuanced analyses of how differences between localities can affect children's schooling outcomes. Such an understanding may help us to uncover successful programmes that may be relevant and beneficial to other settings. These advantages of longitudinal and cross-country analyses justify our decision to highlight the evidence gap in the literature on school access around use of longitudinal survey data and cross-country data. The objectives of this paper are to

1. identify gaps in the literature on children's school access using CREATE's zones of exclusion as a framework and
2. discuss how these gaps can be met by using data from HDSS sites within the INDEPTH Network.

The paper is structured as follows. We first present the methods that we used to achieve our research objectives. We describe the process for the literature search, detailing the databases and keywords we used. We then present our findings summarising the publications obtained from our literature search by using the keywords ‘school outcome’, ‘use of longitudinal data’, and ‘cross-country studies’. Following this, we present a discussion of how data from HDSS sites can contribute to narrowing the gaps that we identify through our review. In the conclusion, we summarise the main findings from this review and highlight the policy implications of our research to children's school access in LMICs.

## Methods

We conducted this research in three stages. In the first stage, we performed a systematic literature review of studies using Web of Science, a reference database holding citations for every discipline and world region. We searched for six phrases including: ‘school enrolment’, ‘school attendance’, ‘grade progression’, ‘school dropout’, ‘primary to secondary school transition’, and ‘school completion’. Each search was defined by journal publications in LMICs in Africa, Asia, and Oceania because these are the countries which form the INDEPTH Network. Also, we restricted our search to studies conducted between 1998 and 2013 as the INDEPTH was established in 1998 and the EFA was included in the MDGs in the year 2000. From our literature search, 1,481 references were returned: grade progression (418 references), primary to secondary school description (329 references), school attendance (274 references), school enrolment (234 references), school completion (143 references), and school dropout (83 references). Of the 1,481 references returned, only 132 were relevant to our focus on school access. In the second stage, we reviewed the 132 references and summarised them according to our key phrases or school outcomes. Finally, we made note of all studies that used longitudinal data sources and studies that used data from more than one country.

## Results

This section presents our results from the literature review. We first present the publications which we found to be relevant to our search; we summarise findings according to publications which focused mainly on one of the six school outcomes that we searched and those which explored more than one of the school outcomes (Table 2). Subsequently, the findings from our analysis of studies using longitudinal data and cross-country data are presented, respectively.

**Table 2:** All references obtained from search in Web of Science by school outcome

Phrase searched	Total relevant publications returned from keyword search	Total publications which focused on outcome
School enrolment	71	49
School attendance	24	19
Grade progression	3	0
School dropout	24	19
Primary to secondary school transition	3	3
School completion	7	4
More than one outcome	n/a	38

From our search, a total of 132 references were found to be relevant to children's schooling as framed within CREATE's zones of exclusion. Having reviewed these references, ‘school enrolment’ was the most analysed school outcome (71 publications). More than half of the studies which analysed school enrolment as an outcome focused mainly on children's enrolment (49 out of 71 publications). ‘School attendance’ (24 publications) and ‘school dropout’ (24 publications) were the second most analysed outcomes. As with school enrolment, the majority of studies published on school attendance and school dropout were focused singularly on exploring these outcomes – 19 out of the 24 publications for school attendance and 19 out of the 24 publications for dropout were focused mainly on analysing children's attendance and dropout, respectively. The least studied school outcomes were ‘grade progression’ (3 publications) and ‘primary to secondary school transition’ (3 publications). Few studies have also been conducted on ‘school completion’ (7 publications). All the publications that we reviewed on ‘primary to secondary transition’ analysed only this outcome in the study. In contrast, all the publications we reviewed for grade progression did not solely focus on exploring children's progression between grades; they analysed other outcomes such as dropout, completion, and school entry.

Between 1998 and 2013, journal publications on longitudinal studies which explored children's school outcomes in LMICs were scarce (Tables 3 and 4). Table 3 shows publications that used longitudinal data and analysed one of the school outcomes that we searched. Table 4 also shows publications which used longitudinal data but where more than one outcome was analysed. Of the 132 that we reviewed, 33 made use of longitudinal data. In Table 3, we see that the use of a longitudinal data source has been most frequent among studies where school enrolment is the main outcome variable (10 publications). Five of the 19 studies on school dropout made use of longitudinal surveys compared to 3 of the 19 studies on school attendance. The publications on school completion and transition from primary to secondary school had one study each where longitudinal data were used. In Table 4, 13 studies (of the 38 studies that analysed more than one school outcome) were found to have made use of longitudinal data.

**Table 3:** Publications using longitudinal data which explored mainly one school outcome arranged by the source of data that was used, country in which data were collected and reference for the publication

Outcome	Data source	Country	Reference
School enrolment	Longitudinal study (2000–2003) Kanchanaburi Demographic Surveillance System (2000–2004) African Centre for Health and Population Studies Panel data (2004–2007) Panel data from KwaZulu-Natal Income Dynamics Study (1993–1998) APHRC household data (2000–2005) APHRC household data (2005–2009) APHRC 2005 schooling history data APHRC household data (2005–2009) Ethiopian Environmental Household Study (2000–2007)	Thailand Thailand South Africa Kenya South Africa Kenya Kenya Kenya Kenya Ethiopia	Jampaklay (6) Mahaarcha and Kittisuksathit (7) Case et al. (8) Nishimura and Yamano (9) Handa and Peterman (10) Ngware et al. (11) Oketch et al. (12) Oketch et al. (13) Oketch et al. (14) Lindskog (15)
School attendance	Panel household survey (1991–1994) PASADA community faith-based agency Young Lives household survey	Tanzania Tanzania India	Ainsworth et al. (16) Ng’ondi (17) Woodhead et al. (18)
School dropout	Kanchanaburi Demographic Surveillance System (2001–2004) Community and School Studies data (2007–2009) 2009–2011 panel data set Longitudinal school-based dropout study (1999–2001) Individual-level data (2008–2009)	Thailand Bangladesh China Kenya Cambodia	Korinek and Punpuing (19) Sabates et al. (20) Yi et al. (21) Nyambedha and Aagaard-Hansen (22) No et al. (23)
Primary to secondary school transition	Household survey, Uttar Pradesh	India	Siddhu (24)
School completion	Nang Rong Social (1984, 1994, 2004)	Thailand	Piotrowski and Paat (25)

APHRC (African Population Health Research Centre) collects data in an urban demographic surveillance system in Nairobi, Kenya: Viwandani and Korogocho (slums); Jericho and Harambee (non-slum).

**Table 4:** Publications which used longitudinal data and analysed multiple school outcomes arranged by the source of data that was used, country in which data were collected and reference for the publication

School outcome	Data source	Country	Reference
Attendance and highest grade attained	Household survey (2005–2007)	Ethiopia	Belachew et al. (26)
Enrolment in grade 1, grade progression, primary school completion	Birth-to-Twenty cohort panel study	South Africa	Fleisch and Shindler (27)
Enrolment, attendance, school entry, grade repetition	Administrative data from (2000–2005)	Chile	McEwan (28)
School participation – enrolment, dropout	Panel data (1998–2002)	Kenya	Evans and Miguel (29)
Attendance and enrolment	School panel data 2003–2004	India	Afridi (30)
Enrolment, years of education completed	Household survey (1995–2004–2005)	Burkina Faso	Kazianga (31)
Enrolment and completion	Demographic surveillance area KwaZulu-Natal (2000–2004)	South Africa	Case and Ardington (32)
Dropout and enrolment	Data from birth histories and birth history	South Africa	Grant and Hallman (33)
Attendance and dropout	Intervention study (2008–2009)	Malawi	Pridmore and Jere (34)
Grade of dropping out, grade of enrolment	Gansu Survey of Children and Families (2000–2004)	China	Zhao and Glewwe (35)
Grade repetition; grade attainment	Senegal Household Education and Welfare (1995–2003)	Senegal	Glick and Sahn (36)
Grade progression, school mobility, age at school entry	Birth-to-Twenty cohort study	South Africa	Ginsburg et al. (37)
Dropout, age-in-grade-progression, and repetition	Education Management Information Systems	South Africa	Motala et al. (38)

There was some variation in the data source of the longitudinal surveys and the countries in which the surveys were conducted. The surveys were more likely to have been conducted in countries in sub-Saharan Africa (21 publications) and Asia (11 publications). The most frequently studied countries were South Africa (7 publications) and Kenya (7 publications). The data sources from the studies on these countries were similar. Four of the seven studies on South Africa used data from the Demographic Surveillance Area in KwaZulu-Natal (8, 10, 32, 33); two used data from the Birth-to-Twenty cohort panel study (27, 37); and the remaining study used data from the Education Management Information System (38). For the studies on Kenya, data from Nairobi's Demographic Surveillance System sites collected under the African and Population Health Research Centre's Education Program were the most frequent source (11–14). Three of the studies however used data from elsewhere: 1) Evans and Miguel (29) used panel data collected from a Pupil Questionnaire and Tracking survey between 1998 and 2002 in Busia district; 2) Nyambedha and Aagaard-Hansen (22) analysed data from a school-based dropout study in Western Kenya; and 3) Nishimura and Yamano (9) made use of panel data collected from household community survey in rural Kenya.

Other countries such as Thailand (four publications), India (three publications), China (two publications), Ethiopia (two publications), and Tanzania (two publications) were also studied using longitudinal surveys. Three of the four studies which performed longitudinal analyses for Thailand used the same data from the Demographic Surveillance System site in Kanchanaburi Province (6, 7, 25). The studies on India all used different data sources: one study used a household survey from Uttar Pradesh (24); another study used data from the Young Lives household survey (18); and the last study used school panel data (30). The studies on China (21, 35), Ethiopia (15, 26), and Tanzania (16, 17) also used data from different sources.

Very few of the studies that we reviewed performed cross-country analyses (Table 5). Of the 132 publications that we reviewed, 17 performed cross-country analyses. Studies which explored school enrolment (*n =*6) as the main outcome had the most number of cross-country publications followed by those on dropout (*n*=3) and attendance (*n =*2). Countries in sub-Saharan Africa were the most likely to be included in comparative studies: 13 of the 17 studies were focused only on countries in the sub-Saharan context. Among the remaining studies, four were focused on LMICs more broadly (39–41) with one of these studies analysing data from low-income countries only (42).

**Table 5:** Publications that used data from more than one country arranged by the source of data that was used, country in which data were collected and reference for the publication

Outcome	Data source	Country	Reference
Attendance	World Bank Unit record household data sets Demographic and Health Survey (DHS)	15 African countries 30 countries in Africa	Kakwani et al. (43) Longwe and Smits (44)
Enrolment	Cross-sectional surveys DHS and Integrated Household Survey (IHS) Case study DHS Case study: Ministry of Education, United Nations, interviews, survey Case studies: interviews and observations of schools	Malawi and Kenya 34 sub-Saharan African countries Ghana, Nigeria and Togo 21 poor countries Guinea and Ethiopia Jamaica, Kenya, Tanzania, Ghana, Indonesia, Pakistan	Schafer (45) Smith-Greenaway and Heckert (46) Tuwor and Sossou (47) Filmer (42) Colclough et al. (48) Heyneman and Stern (41)
Dropout	National Survey of Adolescents DHS DHS	Burkina Faso, Uganda, Ghana, Malawi Burkina Faso, Cameroon, Ivory Coast, Guinea, Togo 20 countries in sub-Saharan Africa	Biddlecom et al. (49) Lloyd and Mensch (50) Melhado (51)
Completion	Multiple Indicator Cluster Survey; DHS	Africa	Lloyd and Hewett (52)
Multiple outcomes	DHS Armed conflict data set of the international peace research institute DHS DHS Education Management Information Systems	Kenya, Malawi, Nigeria, Tanzania, Uganda, and Zambia 43 countries in Africa Developing countries Global Sub-Saharan Africa	Lewin and Sabates (53) Poirier (54) Grant and Behrman (40) Filmer and Pritchett (39) Lewin (4)

The majority of the data used in these studies originated from cross-sectional household surveys. The Demographic and Health Survey (DHS) was the most frequently used data source: 8 of the 17 studies used the DHS for analyses. The Integrated Household Survey and Multiple Indicator Cluster Surveys were also used (46, 52). These surveys, like the DHS, are large-scale surveys designed to be national representative which are used to collect demographic, health, poverty, and education indicators in LMICs. Biddlecom et al. (49) also used a large-scale survey (i.e. National Survey of Adolescents) although this survey is administered only in four countries in sub-Saharan Africa: Ghana, Burkina Faso, Uganda, and Malawi. Some studies used a case study approach, triangulating different sources of data, for their research (41, 48, 47). Among the three remaining studies, one used data from Education Management Information Systems (4); another used the World Bank Unit record household data sets (43); and the last made use of data from the armed conflict data set of the international peace research institute (54).

## Discussion

The first objective of this paper has been to identify gaps in studies on children's school access in LMICs. The main gaps which we have identified can be summarised as such:

1. Grade progression, primary to secondary school transition, and completion were the least studied school outcomes.
2. Around a quarter of studies (33 out of 132 publications) in our review used data collected over time.
3. Studies which used longitudinal data were more likely to have been conducted in South Africa, Kenya, and Thailand. The data from these studies were collected mainly from Demographic Surveillance System sites.
4. Just over one-tenth of studies (17 out of 132 publications) in our review performed cross-country analyses.
5. More than two-thirds of the cross-country analyses (13 out of 17 publications) were focused only on countries in sub-Saharan Africa. The most frequently studied countries were Ghana, Malawi, and Uganda.
6. Large-scale cross-sectional surveys were most frequently used to perform cross-country analyses; the DHS was the main data source.

Data from HDSS sites operating within the INDEPTH Network can contribute to narrowing the gaps which have been highlighted in this review. The INDEPTH Network oversees and coordinates multisite research activities in 52 HDSS sites in 20 LMICs in Africa, Asia, and Oceania (Table 6). Data on children's school attendance, including the grade and level of education being attended, are routinely collected among the population under surveillance within the HDSS sites. Children's school data are often enumerated at the beginning of the academic school year. These data can therefore be compared across years to observe whether a child returns to school and which grade a child attends from year to year. Where data are collected more than once a year, as in Ifakara (Tanzania) and Ouagadougou (Burkina Faso) for instance, we can observe disruptions in children's schooling during the academic year helping us to understand access beyond simple enrolment. That is, the data can be used to answer process-driven questions such as what happens to children when they enter school; how do children move from one grade to the next; and how do they transition from one level of education to the next. Exploring these questions can contribute to narrowing the deficit in studies on grade progression, primary school completion, and primary to secondary school transition.

**Table 6:** Health and Demographic Surveillance System sites within the INDEPTH Network arranged by continents

	Africa	Asia	Oceania
Country and HDSS	Burkina Faso: Ouagadougou; Nouna; Sapone; Kaya; Nanoro Cote D’Ivoire: Taabo Ethiopia: Gilgel Gibe; Kersa; Butajira; Dabat; Kilite Awlaelo The Gambia: Farafenni; West Kiang Ghana: Navrongo; Dodowa; Kintampo Guinea Bissau: Bandim Kenya: Kisumu; Kombewa; Mbita; Kilifi; Nairobi Malawi: Karonga Mozambique: Chokwe; Mahinca Nigeria: Nahuche; Cross River Senegal: Bandafassi; Niakhar; Mlomp South Africa: ACDIS, Agincourt; Dikgale Tanzania: Ifakara; Rufiji; Magu Uganda: Rakai; Iganga/Mayuge; Kyamulibwa	Bangladesh: Matlab; Chakaria; Bandarban India: Ballabgarh; Birbhum; Vadu Indonesia: Purworejo Thailand: Kachanaburi Vietnam: Chililab; Dodolab; Filabavi	Papua New Guinea: Wosera; PIH

The longitudinal design of the HDSS offers significant potential for studying children's schooling outcomes. The operation of the HDSS allows children to be continuously observed and tracked from the year they enter school. This provides rich data that can be used to perform detailed analyses of household schooling decisions over time. Information is also collected at the household and community levels. At the household level, questions are administered on socio-economic and demographic characteristics of the household. At the community level, information is available on school supply as well as type of school, and access to infrastructure, services, and amenities. Data collected at the household level make it possible to observe how changes within the home can affect decisions to send a child to school. Similar analyses can be applied to understand how changes within communities can affect schooling outcomes.

The longitudinal setup of the HDSS also enables us to observe how educational programmes and policies can affect children's schooling. Since 2000, governments in LMICs have introduced a series of measures to expand access, such as school feeding policies, girl-friendly policies, and capitation grants (55, 56). Often, however, these policies are assessed at a national level using large-scale, cross-sectional surveys to estimate enrolment ratios and levels of attainment (40, 53). Using the HDSS sites, it is possible to observe to what extent UPE policies affected children's schooling behaviour and analyse how children progressed in the school system once they entered. It is also possible to compare within countries (for countries with multiple HDSS sites) how response to education policies and programmes varied between localities. The longitudinal structure of the HDSS data can therefore make a significant contribution to educational studies in LMICs by enabling us to observe change over time and explore the temporal sequence of events.

The diversity of countries in the INDEPTH Network presents another way in which data from HDSS sites can make a contribution to educational studies in LMICs. As noted above, there are 20 countries within the INDEPTH Network in which there are 52 HDSS sites. The majority of the HDSS sites are in sub-Saharan Africa (39 out of 52 sites); there are 11 HDSS sites in Asia and 2 HDSS sites in Oceania. In sub-Saharan Africa, the HDSS sites are located in 14 countries; in Asia they are in 5 countries; and in Oceania the 2 HDSSs are located in the same country. The countries in sub-Saharan Africa include Burkina Faso, Cote D'Ivoire, Ethiopia, The Gambia, Ghana, Guinea Bissau, Kenya, Malawi, Mozambique, Nigeria, Senegal, South Africa, Tanzania, and Uganda. In Asia the countries are Bangladesh, India, Indonesia, Thailand, and Vietnam; in Oceania there is Papua New Guinea. The majority of comparative studies which have so far been conducted have focused on countries in sub-Saharan Africa, namely Ghana, Uganda, and Malawi. The countries within the INDEPTH Network are diverse and can be used to form comparisons between African and Asian countries as well as with Papua New Guinea. Even within the same continent, there are many countries which so far have been little explored. In the sub-Saharan context for instance, so-called Francophone countries have been less represented in the literature. Children's school access can be compared between these countries and the others in the sub-region as well as with those in Asia.

Among the cross-country studies that we reviewed, cross-sectional surveys designed to be national representative were mainly used with the DHS being the most frequently used survey. One of the constraints of using the DHS for studying education outcomes is that the survey does not collect information on school supply variables. Therefore, apart from Filmer's (42) study which used a special round of the DHS that had collected information on distance to school, none of the studies could account for school supply variables. Another limitation of the DHS is that analyses cannot be performed to understand how patterns and trends in access to school change over time. The most common theme among the cross-country studies that we reviewed was to demonstrate levels of school enrolment through univariate and bivariate analyses (controlling for sex of the child, household poverty, and area of residence). Data from the HDSS sites can contribute to narrowing these gaps by developing more complex and robust models which account for both supply and demand variables. These models can be applied across multiple HDSS sites between countries to assess variations in the factors which affect children's schooling. Additionally, longitudinal models can be developed to evaluate how the determinants of children's schooling outcomes have changed overtime. Assessing change in the determinants of schooling outcomes is justified by the need to target resources more efficiently to areas which have the strongest impact on access.

Surveys conducted at a national level were more frequently used in the cross-country studies. The HDSS sites in contrast are focused often on smaller geographic and administrative regions and uniquely follow marginalised populations such as those in remote rural areas or poor urban informal settlements. Children living in marginalised populations such as urban informal settlements or rural communities have the least access to school (13, 14, 48). These localities are often resource deprived, lacking access to school infrastructure, particularly schools of good quality (57, 58). In these populations, children from poor households and girls are confronted with severe barriers to enter, progress, complete primary school, and transition to secondary school (53, 59, 60). There are few studies which utilise survey data over time to undertake enquiries as to how access among marginalised populations has changed over time and how changes within these contexts affect changes in children's schooling behaviour. Data collected at INDEPTH HDSS sites can contribute to narrowing this gap in the literature. Also, as well as forming comparisons between countries, analyses can be performed on multiple HDSS sites within countries as has been done by studies which have used the Nairobi HDSS (12–14). The emphasis is to uncover how variations both between and within countries can influence a households’ decision-making process to invest in a child's education over time. The location and size of the population under surveillance within HDSS sites therefore offer yet another opportune advantage to conduct more nuanced and detailed comparative analyses.

## Conclusions

The gaps which we have identified through our literature review suggest a significant role for longitudinal data in LMICs to explore educational outcomes beyond school enrolment and attendance. As we move towards a post-2015 development agenda, a broader conceptualisation of school access is likely to become more relevant, demanding a focus away from a dichotomous understanding of school access to one where it is understood as a continuum, a process in which children enter, remain, progress, complete primary school, and transition to higher levels of education. Adopting this alternative approach to understanding school access implies a significant role for studies conducted over time in future research. Longitudinal studies can be useful for observing children's school access as a continuum. Here, data collected repeatedly through sites within HDSS sites can make a contribution to better understand those school outcomes which have been little explored in educational studies in LMICs. Furthermore, the HDSS sites operate in populations which have been found to be the most marginalised in-school access, namely in rural and poor urban areas. The data collected from these sites can be used as evidence to design more targeted policy initiatives for improving participation and retention rates among children in deprived populations.
